# Sport Community Involvement and Life Satisfaction During COVID-19: A Moderated Mediation of Psychological Capital by Distress and Generation Z

**DOI:** 10.3389/fpsyg.2022.861630

**Published:** 2022-04-14

**Authors:** Juho Park, Jun-Phil Uhm, Sanghoon Kim, Minjung Kim, Shintaro Sato, Hyun-Woo Lee

**Affiliations:** ^1^Department of Health and Kinesiology, Texas A&M University, College Station, TX, United States; ^2^Graduate School of Sport Sciences, Waseda University, Tokyo, Japan

**Keywords:** sport community involvement, psychological capital, life satisfaction, distress, generation Z, COVID-19

## Abstract

How can sport community involvement influence life satisfaction during a pandemic? Self-expansion theory posits that individuals seek to gain resources such as positive interpersonal relationships for growth and achievement. By considering psychological capital (PsyCap) as a dispositional resource intervening between sport community involvement and life satisfaction, we examined an empirical model to test the chain of effects. Based on the stress process model, distress and generational group (Generation Z vs. others) were tested as moderators. Participants (*N* = 233) responded to the scale item questionnaire for model assessment. Supporting the hypothesized relationships, the model was supported with a significant moderated-moderated mediation. The mediation effect of PsyCap was stronger when distress level was lower and such interaction effect was amplified for Generation Z (Gen Z). Whereas the global sport communities and Gen Z were found to be more particularly vulnerable to COVID-19, our findings suggest that there are psychological pathways for fans to maintain their resilience. It is foremost imperative to lower the stress level of sport fans for their community involvement to positively affect life satisfaction. Gen Z were more stressed during the pandemic but individuals who managed to cope with stress were able to leverage community involvement to boost positive psychological resources. Acknowledgment of these effects brings implications for better management strategies and provides avenues for new research.

## Introduction

Sport organizations and Generation Z (Gen Z) took a big hit from the COVID-19 pandemic. Restrictions on social gatherings led to cancelling sporting events (Woodford and Bussey, 2021) and Gen Z faced reduced job opportunities and lower payments (Glasper, 2020). Sport fan communities around the globe are currently challenged to seek strategies for returning to normal after the pandemic. Whereas sport organizations provide a unique context that exemplifies organizational vulnerabilities to COVID-19, a large body of research suggests positive effects of sporting experiences resilient to such hardship.

The benefits of sports to society have been widely recognized for many years (Coalter, 2007). Individuals can improve their physical and psychological well-being by participating in community sports, and they can also gain at both individual and social levels by joining fan clubs that support certain athletes or teams (Vail, 2007). Social connections among members of a fan community have positive psychological effects by reinforcing a person’s sense of camaraderie and identification, as well as creating a sense of belonging and care, all of which can improve overall life satisfaction (Wann et al., 2015).

Participating in organized sport consumption activities can also help improve psychological well-being by allowing individuals to build up personal-psychological capital (also known as PsyCap). In other words, engaging in community activities can lead to positive appraisals of life circumstances and accentuate the potential for personal achievement, which in turn can derive a sense of fulfillment across a variety of life domains (Bockorny and Youssef-Morgan, 2019). While the concept of PsyCap has received considerable attention in the organizational behavior literature in general, there has not been much research on how this concept could be applicable to benefit the subjective well-being of sport fans.

Foremost, there is a clear gap in the literature examining sport community involvement as an antecedent of PsyCap. Whereas several researchers investigated the value of psychic income and community welfare effects of sports (e.g., Warner and Dixon, 2013; Ouyang et al., 2020), there is a limited empirical study examining the association of sport community involvement with PsyCap. In addition, while there are evidences indicating a positive relationship between job involvement and PsyCap (e.g., Demir, 2018; Rani and Chaturvedula, 2018), the role of involvement in a non-occupational community setting gained little attention. The current investigation expands the boundary conditions of the stress process model (Pearlin et al., 1981) by incorporating self-expansion theory (Aron and Aron, 1996) into the PsyCap literature as we test whether perceptions of community interactions can improve positive psychological resources.

Although previous studies on PsyCap have been conducted in industrial–organizational contexts, its potential to play an important role within the context of sports fan communities cannot be ignored. In most PsyCap organizational behavior studies, scholars have focused on participants’ main occupations (e.g., employees, students) not in non-occupation settings. The sports fan community can serve to improve participants’ self-confidence and self-actualization regardless of their main occupation, making it an important vehicle for self-development and positive psychology (Doyle et al., 2016). A study on involvement in the sport fan community may provide important implications for PsyCap literature, as PsyCap research on leisure activities and relevant phenomena such as the sports fan community has been far ignored. Furthermore, we attempted to address an imperative question that sport management practitioners are currently facing: How can professional sport organizations better understand the psychological state of the younger generation facing the pandemic?

In particular, Gen Z has recently received scholarly interest due to its unique personality and organizational behavior that is different from previous generations (Puiu, 2016; Cilliers, 2017). Gen Z members, in general, enjoy forming bonds with others and seeking new ideas or experiences through such relationships, as well as engaging in community activities (Seemiller and Grace, 2017). However, they are relatively vulnerable to stress as they are not resilient under pressure (American Psychological Association, 2018; Cartwright-Stroupe and Shinners, 2021). Nevertheless, Gen Z has received very little attention in the sport fan community context.

Given that Gen Z members prefer to be in the company of others and are prone to stress (American Psychological Association, 2018), they are likely to face exceptional adversity during the COVID-19 pandemic, which strains and disrupts various restorative life activities. Regarding the positive social and personal benefits that sport fans’ community engagement may provide, investigating the effects of involvement in a sport community on Gen Z members under stressful conditions can provide useful insights into explaining their psychosocial outcomes.

Therefore, in this study, we investigated how Gen Z members’ sport fan community involvement can positively affect their life satisfaction, under pandemic-induced stressful circumstances. Using self-expansion theory and the stress process model, we incorporated PsyCap as a mediator between community involvement and life satisfaction, as well as how stress and belonging to Gen Z affects this relationship.

## Literature Review and Hypothesis Development

### Sport Community Involvement and Life Satisfaction

The concept of involvement has been defined in several ways: As a person’s subjective assessment of how one concerns and cares for, and perceives the importance, personal relevance, and significance of, an attitude (Zaichkowsky, 2012). Involvement, as an activated attitude, affects one’s motivational state of mind regarding to an object or activity (Mittal and Lee, 1989) and elicits engagement in behaviors to accomplish relevant objectives and goals (Poiesz and de Bont Cees, 1995). The operational definition of involvement has been widely used to better understand people’s motives and behaviors in a variety of settings (e.g., education, leisure, work; Zaichkowsky, 2012). Studies on involvement have been extended to comprehend why people get involved in certain activities (e.g., participating in sports; attending sporting events), especially given the fact that community activities have been recognized to positively improve an individual’s psychological state.

A major feature of community involvement is in how people’s interest and personal importance attached to such engagement and participation affects their mental state. Moore et al. (2006) showed that assessing involvement can help better understand and forecast the advantages for both individual (e.g., pleasure, enjoyment, a sense of belonging) and community (e.g., social capital) levels. Numerous researchers have found that community involvement generates positive psychological states including personal well-being (e.g., Gorrell, 2001; Irvine and Warber, 2002; McMahon et al., 2004). Addressing the inherent need to socially engage with others and forge bonds, participation in community activities can positively impact a person’s subjective well-being (Atkinson et al., 2020).

In the spectator sport setting, fans form a community around the team they support and, through this specific community, participate and engage in a variety of fan activities (e.g., attending games, participating in team events, cheering for the team). While researchers have extensively investigated involvement in various sport settings (e.g., sport participation, Funk et al., 2007; viewership, Bee and Havitz, 2010), there has been little study particularly focused on sport fan communities. In this study, we operatively defined sport community involvement as the degree of belonging that followers of sport teams feel regarding their fan communities and the importance they place on participating in relevant fan activities. Further, we highlight self-expansion theory (Aron and Aron, 1996), which has been widely used to examine the relationship between community involvement and life satisfaction, as a useful tool for better understanding the values of sport community involvement.

Self-expansion theory was developed to explain the drive that impacts affection, cognition, and behavior in close relationships (Aron and Aron, 1996). This theory focuses on the individual’s inner motive to grow through acquiring resources that help them achieve their goals (Aron et al., 2005). Self-expansion can take place in a strong relationship that provides opportunity to grow, ultimately resulting in high degrees of positive affect (Aron and Aron, 1996). Through the opportunities for inner growth provided by communal activities, community membership, and involvement, people can attain personal and social goal through positive interpersonal interactions that generate positive affects (McLaughlin-Volpe et al., 2005).

People tend to receive more opportunities for self-expansion through socially engaged experiences within their community, which ultimately leads to greater life satisfaction. Kara and Sarol (2021) articulated that involvement in group activities lead to setting a specific and feasible goal, promoting regular involvement in the activity, and fulfilling the desire for personal growth and social bonds. Being involved in a certain activity triggers a process of experiences where people tend to seek the meaning of the activity, give meaning to their lives, and further consider how it fits into their lifestyle, which may in turn foster positive life evaluations (Sato et al., 2016). With reference to the importance of community involvement in one’s positive affect, we can reasonably expect sport community involvement to have a positive influence on the life satisfaction of sport fans. Therefore, we established the following hypothesis:

*Hypothesis 1*: Sport community involvement will positively affect fan’s life satisfaction.

### Mediating Role of Psychological Capital

Compared to others, people seeking for self-expansion are more likely to obtain additional resources in order to accomplish their own objectives (Aron and Aron, 1996). Among various available resources, Jurek and Besta (2021) highlighted the importance of enhancing an individual’s PsyCap in order to evoke positive attitudes and behaviors in the organizations to which they belong. PsyCap refers to the developmental capacity of individuals including state-like and motivational cognitive constructs such as hope, efficacy, optimism, and resilience (Luthans, 2002). PsyCap is highlighted as one of the core constructs in the positive organizational behavior perspective for its significance in individual growth from the actual self to the possible self (Luthans et al., 2007). Within organizational settings (i.e., business and management), PsyCap has been considered a prominent factor for employee growth, organizational behavior outcomes (e.g., job performance), and well-being (Avey et al., 2010; Newman et al., 2014). Considering such positive aspects in the sporting context, the role of PsyCap in one’s accomplishments and well-being has been adapted and empirically examined across different settings with various targets such as coaches (Kim et al., 2017, 2019), sport administrative employees (Kang et al., 2021; Kim et al., 2021), student-athletes (Kim et al., 2020), and residents of sporting event host cities (Sato et al., 2020).

Examining the mediating role of PsyCap within the mechanism that underlies the influence of a specific life domain (i.e., sport community) augmenting life satisfaction can provide a deeper understanding of the process of enhancing sport fans’ life satisfaction. Wright et al. (2002) proposed that people want to be a part of certain communities because it allows them the chance to expand their horizons and reach personal growth and development goals. In terms of how to improve one’s PsyCap, Tu (2020) maintained that facilitating social relationships among people plays a crucial role in leisure participants’ psychological development. These results suggest that sport community involvement can contribute to an individual’s growth and development. Specifically, individuals can gain greater confidence in dealing with challenges when they receive support from other members in their community (i.e., efficacy), allowing them to discover new ways to achieve their goals (i.e., hope). Also, a sport fan community member’s optimism and resilience can be strengthened by their belief that there will be support and help from other group members when they face difficulties, allowing them to think positively about the future instead of becoming frustrated (Sagi et al., 2021).

PsyCap is closely associated with subjective well-being, which can be defined as “a person’s cognitive and affective evaluations of his or her life” (Diener et al., 2002, p. 63). While subjective well-being is an umbrella-like construct that encompasses a wide range of positive psychological responses, it has been used interchangeably with life satisfaction (Diener et al., 1999; Gupta and Shukla, 2018). There is numerous empirical evidence demonstrating that PsyCap can exert a positive influence on life satisfaction (e.g., Choi and Lee, 2014; Bockorny and Youssef-Morgan, 2019). This is because PsyCap can serve as (1) cognitive resources and a reservoir from which members of the community can draw to impact individuals’ well-being, and (2) an avenue for boosting people’s immunity to stressors or even shaping the ways they appraise and define events (Avey et al., 2010).

PsyCap equips people to reframe events as motivational challenges rather than debilitating threats, in turn helping them to gain a sense of fulfillment or satisfaction (Riolli et al., 2012). For example, Park et al. (2004) argued that individuals with a high level of efficacy and hope tend to have a more positive view about the future and, as a result of this belief, they have a positive persona that helps them maintain a more positive attitude in their own lives. Furthermore, resilience and optimism are positively associated with adaptive coping responses and negatively correlated with negative emotions in adversity, which in turn can impact the subjective well-being of an individual (Andersson, 1996). To sum up, four sub-constructs of PsyCap (i.e., efficacy, hope, optimism, resilience) synergistically enhance one’s life satisfaction.

The function of PsyCap resources assisting individuals to interpret situations in a positive and beneficial manner builds a case for the mediating role of PsyCap between the effect of sport community involvement on members’ subjective well-being. Based on the self-expansion theory and existing findings, PsyCap can help members of sport communities feel motivated, energized, and adaptable when facing adversity (e.g., a global pandemic). As a result, two direct effects among three key factors are investigated in the current research (i.e., from sport community involvement to PsyCap; from PsyCap to life satisfaction), leading to the following hypothesis:

*Hypothesis 2*: Fan’s PsyCap will mediate the relationship between sport community involvement and life satisfaction.

### Moderating Effects of Distress

Numerous recent studies have focused on individuals’ distress levels in facing a global pandemic (e.g., Ang et al., 2021). As a generic term, distress has been linked to mental tension and or strain and is often perceived as a nonspecific bodily response to a stimulus. The subjective feeling of distress is triggered by a stimulus (i.e., a stressor) or environmental demands (Krannich et al., 1988). In the field of spectator sport, distress has been studied mostly in terms of its effects on fans’ well-being, particularly given the onset of the COVID-19 pandemic as a stressor. Studies found that the pandemic has imposed numerous restrictions on sports fans and their communities, putting them under a significant level of stress (Schellenberg et al., 2021). Further, it was evidenced that fans’ anxiety over when they will be able to return back to participating in fan activities without worrying about the disease was stressful and perceived as a threat to their self-identity (Schellenberg et al., 2021).

The stress process model, commonly employed to better understand the effects of distress on an individual (Pearlin et al., 1981), has been a primary sociological lens for comprehending the relationships among stressors, individual resources, and mental health. In this model, stressors are defined as “the broad array of problematic conditions and experiences that can challenge the adaptive capacities of people” (Pearlin, 2010, p. 208). In other words, the stressors can be viewed as external forces hindering one’s ability to function normally, thus constituting a primary attribute of poor mental health (Wheaton et al., 2013). Based on the stress process model, external stressors can exert a direct negative effect on an individual’s mental health (e.g., depressive symptoms). Additionally, the stressors negatively affect personal resources (e.g., self-esteem and self-mastery) and ultimately their mental health.

In facing the COVID19 pandemic, the aforementioned relationships can be differed according to fans’ levels of distress caused by a lack of in-person social interactions through participation in sport community activities. Such people are more inclined to satisfy their intrinsic personal and social needs through their perceived involvement in sports communities and, in doing so, their self-improvement is more dependent on the perceived social support from and interaction with others (Aron and Aron, 1996; McLaughlin-Volpe et al., 2005). The pandemic has limited the ability of those heavily involved in the sports community to engage in community activities, and the resulting stress can affect their PsyCap and lead to a consequent deficit in life satisfaction. Conversely, the effect of such stress may be relatively smaller for fans who are less involved in sports community activities, as they may satisfy their intrinsic desires by other means. In this respect, it is hypothesized that the extent to which an individual can enhance their PsyCap through sports community involvement varies depending on their level of stress. Accordingly, we established the third hypothesis as follows.

*Hypothesis 3*: Distress moderates the mediation effect of PsyCap.

### Moderating Effects of Generation Z

Gen Z refers to those who are born between 1995 and ending around 2010. During this time span, various events occurred such as the advancement of technology, movements regarding social issues (e.g., equality), and an unsteady economy (Turner, 2015; Hampton and Keys, 2017). Exposed to those events, Gen Z relishes socializing with other people, sharing ideas and experiences through community involvement, and influencing other people’s thoughts and behaviors (Turner, 2015; Hampton and Keys, 2017). With those characteristics, Gen Z has become the mainstream of modern society, and thus scholars have actively attempted to understand the features of Gen Z and their impact on various parts of society. For instance, some scholars looked into how the characteristics of Gen Z interplays within the educational system (e.g., Hampton and Keys, 2017; Chicca and Shellenbarger, 2018) and others came to learn how the life experiences of Gen Z differ from others in the context of tourism (Robinson and Schänzel, 2019) and public policies (Giachino et al., 2022).

Among the various characteristics, Gen Z may be more prone to stress and its negative consequences as a result of the social circumstances surrounding them. For instance, Gen Z has grown by witnessing and naturally being exposed to the advancement of cutting-edge technology (Seemiller and Grace, 2017), which allowed them to obtain a lot of information in a short amount of time (Hampton and Keys, 2017). It also, however, engendered unintended consequences. They lack patience and are reluctant to engage in in-depth problem-solving thought processes (Turner, 2015; Hampton and Keys, 2017). Compared to other generations, Gen Z tends to stress out and feel depressed more easily with more severe consequences (Turner, 2015). Overall, studies indicate that Gen Z possesses unique characteristics, which may influence a variety of psychological and behavioral outcomes in a variety of situations. This also highlights the need to understand the numerous viewpoints regarding Gen Z that occur in distinct contexts.

As emphasized earlier, the stress process model explains how stressors can exert a stronger negative impact on the mental health of certain people than others (Pearlin et al., 1981). In this sense, Gen Z can be more vulnerable to stressors than other generations when facing the COVID-19 pandemic. Previous studies found that interpersonal or face-to-face social interactions cannot be completely replaced by other forms of social interaction (Turkle, 2011; Turner, 2015). Thus, the COVID-19 pandemic may increase Gen Z’s stress levels by hindering their in-person social interactions through sport community involvement, impacting their mental health. As a result, we postulated that the aforementioned hypothesized relationship (H3) could differ for Gen Z members compared to other generations. Accordingly, the following hypothesis was generated:

*Hypothesis 4*: Gen Z moderates the mediation effect of PsyCap moderated by fans’ distress.

## Materials and Methods

### Participants

We recruited sport fans *via* Amazon Mechanical Turk. Two screening questions were used at the beginning of the survey to verify whether the participants were sport fans or not. Specifically, participants were asked to answer an open-ended question regarding their favorite sport team and indicated their level of identification with the team. Further, we utilized a fraud and bot detection system (i.e., reCAPTCHA) as protection against fraud and abuse in collecting data. We removed invalid responses from the analysis. Of the 331 contacts, a total of 233 responses were deemed usable: 156 males (66.95%) and 77 (33.05%) females. As for Gen Z, 98 survey participants (42.06%) belonged to Gen Z, while 135 participants (57.94%) did not. Gen Z showed higher levels of distress (*M* = 4.58, *SD* = 1.51) compared to non-Gen Z (*M* = 4.07, *SD* = 1.87; *F* = 4.95, *p* < 0.05). In terms of the academic completion levels, 9 participants were high school graduates, 11 participants engaged in some college but had no degree, 9 had an associate’s degree (2 years), 166 had a bachelor’s degree (4 years), 36 had a master’s degree, and two had a doctoral degree. Majority of the respondents were Asian or Pacific Islander (*n* = 138, 59.2%) followed by White or Caucasian (*n* = 56, 24.0%), Black or African American (*n* = 15, 6.4%), Hispanic or Latino (*n* = 12, 5.2%), Other (*n* = 9, 3.9%), and American Indian or Native American (*n* = 3, 1.3%).

### Measures

We adapted items from the previous studies, and their reliability and validity have been adequately assessed. The selected items were revised and reworded for this research, considering content relevance, representativeness, and item clarity. Specifically, we adopted 10 items from the revised personal involvement inventory (RPII), established by Zaichkowsky (1994), to measure sport community involvement. Twelve items from the PsyCap instrument (Luthans et al., 2007) were adopted to fit the context of the sport fan community. Five items relating to life satisfaction were adopted from the study by Pavot and Diener (2008). Finally, 11 items measuring distress symptoms were adopted from the Symptom Checklist-90 (SCL-90) scale (Derogatis and Cleary, 1977). The wording of items is listed in Appendix.

### Statistical Analysis

**Figure 1:**
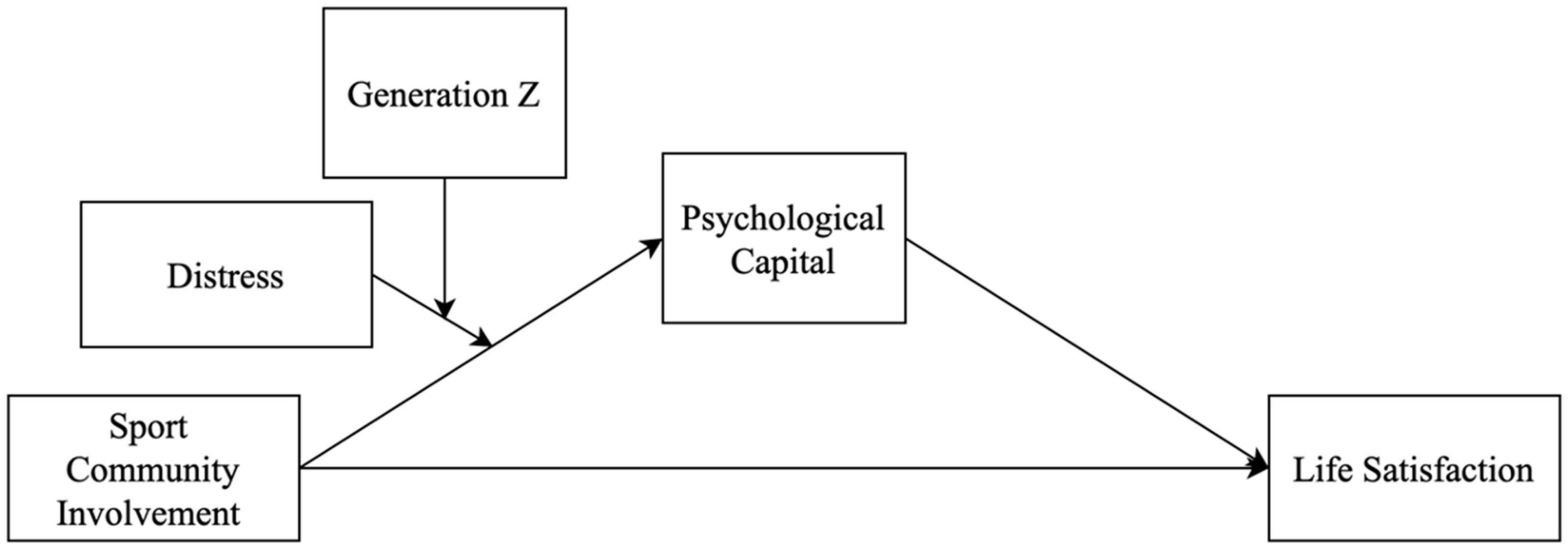
Research model.

The collected data were screened, and the basic assumptions were checked to further analysis. To test our hypotheses, a moderated-moderated mediation model was constructed and tested using PROCESS macro for SPSS (Model 11; Hayes, 2018). Based on the hypothesized relationships, we set sport community involvement as the independent variable, life satisfaction as the dependent variable, PsyCap as the mediator, and distress and generation as a first and second moderator, respectively (see Figure 1). Specifically, in this analysis, we tested the hypothesized direct (H1) and indirect paths (H2) as well as checked conditional indirect effects where the two-way (H3) and three-way interactions (H4).

## Results

Descriptive statistics of study variables are displayed in Table 1, and the path coefficients and confidence intervals are presented in Table 2. Hypothesis 1 proposed that sport community involvement will positively affect life satisfaction, which was not statistically significant (*b* = −0.05, *SE* = 0.03, 95% *CI* = −0.11 to 0.01). However, other results revealed the significant mediation effect of PsyCap in the relationship between sport community involvement and life satisfaction (H2) conditional on the two-way interaction between sport community involvement and distress (H3) and on the three-way interaction of sport community involvement, distress, and the Gen Z (H4).

**Table 1:** Descriptive statistics and correlations for study variables (*N* = 233).

	1.	2.	3.	4.
1. Sport community involvement	1			
2. PsyCap	0.13^*^	1		
3. Distress	−0.41^**^	0.03	1	
4. Life Satisfaction	0.03	0.77^***^	0.15^*^	1
*M*	4.80	5.46	4.28	5.30
*SD*	1.55	0.84	1.74	1.01
Skewness	−0.30	−1.53	−0.59	−1.35
Kurtosis	−1.16	4.04	−1.10	2.62
Cronbach’s *α*	0.97	0.92	0.98	0.86

* *p < 0.05*;

** *p < 0.01*;

*** *p < 0.001*.

**Table 2:** Path estimates.

Predictors	*b*	*SE*	*p*	LLCI	ULCI
DV: PsyCap (*R*^2^ = 0.13)					
Sport community involvement	0.41	0.15	<0.01	0.11	0.71
Distress	0.41	0.17	<0.05	0.07	0.75
Gen Z	−4.40	1.67	<0.01	−7.69	−1.10
Sport community involvement × Distress	−0.07	0.03	<0.05	−0.13	−0.01
Sport community involvement × Gen Z	0.75	0.28	<0.01	0.18	1.31
Distress × Gen Z	0.80	0.31	<0.05	0.18	1.41
Sport community involvement × Distress × Gen Z	−0.13	0.05	<0.05	−0.24	−0.03
DV: Life satisfaction (*R*^2^ = 0.60)					
Sport community involvement	−0.05	0.03	0.07	−0.11	0.01
PsyCap	0.94	0.05	<0.001	0.84	1.04

*DV, dependent variable; SE, standard error; LLCI, lower level of confidence interval; ULCI, upper level of confidence interval*.

The index of the moderated-moderated mediation was statistically significant (*b* = −0.13, *SE* = 0.06, 95% *CI* = −0.25 to −0.01), indicating that the mediation effect of PsyCap on the relationship between sport community involvement and life satisfaction depends on the levels of distress and their generation. Specifically, for Gen Z, the mediation effect of PsyCap moderated by distress was −0.06 (*SE* = 0.03, 95% *CI* = −0.14 to −0.01), while for those who do not belong to Gen Z was −0.19 (*SE* = 0.06, 95% *CI* = −0.31 to −0.09). Based on the result, it can be also explained that the mediation effects of PsyCap moderated by distress were statistically significant regardless of whether the fans belong to Gen Z or not, supporting hypothesis 3. At the same time, the mediation effect of PsyCap moderated by distress changed from −0.06 (*SE* = 0.03, 95% *CI* = −0.14 to −0.01; not Gen Z) to −0.19 (*SE* = 0.06, 95% *CI* = −0.31 to −0.09; Gen Z) conditional on whether fans belong to Gen Z, supporting hypothesis 4.

**Figure 2:**
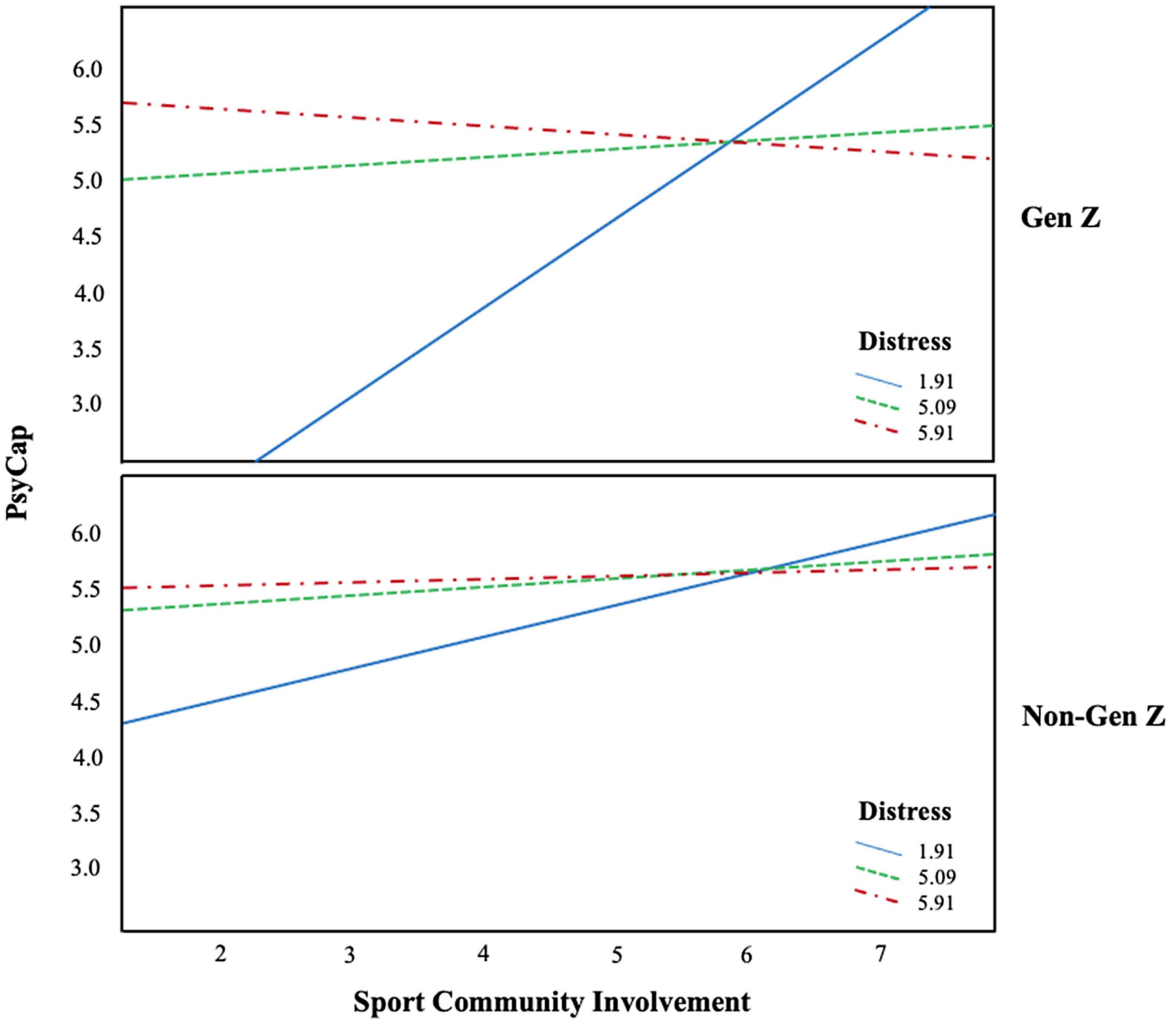
The three-way interaction effect. Solid line represents low distress level at 16th percentile; dotted line represents medium distress level at 50th percentile; and dash-dotted line represents high self-esteem level at 84th percentile.

Taking a more careful look at the three-way interaction results (see Table 3), when the distress level is low (16th percentile), the effect was stronger for Gen Z (*b* = 0.72, *SE* = 0.21, 95% *CI* = 0.35–1.16) compared to those who do not belong to Gen Z (*b* = 0.27, *SE* = 0.11, 95% *CI* = 0.10–53). Next, when the distress is moderate (50th percentile), the effect was significant only for Gen Z (*b* = 0.12, *SE* = 0.05, 95% *CI* = 0.02–0.23), but not for those who do not belong to Gen Z (*b* = 0.06, *SE* = 0.06, 95% *CI* = −0.07–0.16). Lastly, when the distress level is high (84th percentile), the effect was not statistically significant for both Gen Z (*b* = −0.03, *SE* = 0.05, 95% *CI* = −0.13–0.06) and those who do not belong to Gen Z (*b* = 0.01, *SE* = 0.07, 95% *CI* = −0.15–0.12). The three-way interaction results were visualized in Figure 2.

**Table 3:** Conditional indirect effects of sport community involvement on life satisfaction *via* PsyCap.

Distress Level	Generation	Effect	Boot*SE*	BootLLCIs	BootULCI
Low	Non-gen Z	0.27	0.11	0.10	0.53
Low	Gen Z	0.72	0.21	0.35	1.16
Median	Non-gen Z	0.06	0.06	−0.07	0.16
Median	Gen Z	0.12	0.05	0.02	0.23
High	Non-gen Z	0.01	0.07	−0.15	0.12
High	Gen Z	−0.03	0.05	−0.13	0.06

*Distress Level: Low = 1.9 (16th percentile), Median = 5.1 (50th percentile), High = 5.9 (84th percentile); SE: standard error; LLCI: lower level of confidence interval; ULCI: upper level of confidence interval*.

## Discussion

The current study shed light on the underlying psychological processes explaining the impact of sport community involvement on life satisfaction. During the COVID-19 pandemic outbreak, in particular, we expected PsyCap to intervene this process as a positive psychological resource essential to maintaining individuals’ subjective well-being. Moreover, regarding the stress process model and research on Gen Z, we expected this relationship to be moderated by the level of stress where the level of stress would also depend on whether the individual belongs to Gen Z or not. Empirical evidence supported the proposed model by the significant mediation effect *via* PsyCap and significant interaction effects by distress and Gen Z categorization. Previous literature has paid keen attention to the relationship between involvement and life satisfaction or related constructs (Sato et al., 2016; Kara and Sarol, 2021), but explanations of the intervening and dispositional variables have been insufficient. In this sense, the present study can add essential knowledge to the literature by further explaining the conditional processes between sport community involvement and life satisfaction.

The results of the moderated-mediation model indicated that sport community involvement was indirectly linked to life satisfaction through PsyCap. It is important to note that the direct association between sport community involvement and life satisfaction was not apparent when PsyCap was entered in the regression model. This implies the prominent role of PsyCap in explaining life satisfaction, which is consistent with previous literature (Choi and Lee, 2014; Bockorny and Youssef-Morgan, 2019), hence supporting Hypotheses 1 and 2.

Our findings confirm self-expansion theory by highlighting the mediating role of PsyCap. Specifically, results indicate that sport fans can enhance their motivations and goals for self-growth and development by engaging in social activities in their sport communities, which ultimately leads to increasing life satisfaction (Aron and Aron, 1996; Stenseng et al., 2012). The results support the previous evidence suggested by Li et al. (2014) and Huang and Zhang (2021), who presented that PsyCap acts as an important mediator between individuals’ perception of social support from their social network and subjective well-being. Further, our findings broadly support the body of research linking sport community involvement with PsyCap (e.g., Morgan et al., 2019) and PsyCap with life satisfaction (e.g., Bockorny and Youssef-Morgan, 2019). We provide notable evidence confirming a pathway connecting sport community involvement with life satisfaction *via* PsyCap. Whereas previous studies mainly provided evidence linking two of the three key factors (i.e., sport community involvement and PsyCap; PsyCap and life satisfaction), we empirically examined the chain of effects among these factors.

Distress significantly moderated the effect of sport community involvement on PsyCap, supporting hypothesis 3. Sport community involvement affected PsyCap more positively when the distress level was low. It is imperative to consider this disordinal interaction as the effect of sport community involvement became significantly weaker when fans are stressed. As many restrictions have been placed on sports fans and their community involvement as a result of the pandemic, current study results are in accord with recent studies identifying the significant role of stress on fans (Schellenberg et al., 2021). Simultaneously, our results confirm and expand the stress process model (Pearlin et al., 1981), suggesting that such global pandemics or other disasters can act as an external stressor negatively affecting personal-PsyCap, resulting in a negative impact on mental health. Further, we found that Gen Z is experiencing higher levels of distress than others. The stress process model explained that stressors can exert a stronger negative impact on the mental health of certain people than others. In this regard, the demographic cohort information provides additional evidence to support the theoretical explanation and prediction of the stress process model.

The significant moderated-moderated effect further spotlights the importance of distress levels and generation differences based on the three-way disordinal interaction (Figure 2). The mediation of PsyCap showed stronger effects when distress level was low and this interaction effect was more so the case to Gen Z. Visualized in Figure 2, engaging in fan community activities was more impactful for less-stressed Gen Z group. For Gen Z fans, the indirect effect was prominent, especially when their distress level is low and medium (see Table 3). In contrast, for non-Gen Z fans, the indirect effect of sport community involvement through PsyCap was only significant when their distress level is low. It indicated that unless Gen Z’s distress level is notably high, their life satisfaction can be improved by the increased level of PsyCap generated by sport community involvement. Such findings can encourage practitioners and policymakers in sport as the adaptive role of sport community involvement in relation to life satisfaction is further evidenced. However, it is also imperative to note that the role of sport community involvement is not necessarily effective in boosting PsyCap and life satisfaction when individuals are highly stressed out. Scholars need to provide evidence-based solutions based on the findings that it is important to keep the level of distress low for community members, specifically to a greater degree to Gen Z as they are more vulnerable to stress (Turner, 2015).

The findings of this study complement those of earlier studies, as we included distress and generational identity (specifically Gen Z and non-Gen Z) as moderating variables. Although Gen Z has become a major demographic group in society, there has not been adequate research on how their psychological well-being can benefit from sport community involvement. The findings evidence explanations and predictions of how the dynamics of distress, PsyCap, and well-being are organized differently across generations in the context of sport fan community involvement.

This study provides important theoretical contributions by expanding PsyCap’s application beyond job settings. By examining the role of PsyCap in the sport fan community context, we confirmed fan community involvement as an antecedent of PsyCap which is not delineated by participants’ occupations. This study also expands the boundary conditions of the stress process model (Pearlin et al., 1981) by incorporating self-expansion theory into the PsyCap literature. Laying the foundation for better understanding of specific phenomena related to sport fan communities is an arguably significant academic contribution.

Our study also highlights the important role of sport fan community involvement in improving participants’ self-confidence and self-actualization leading to self-development and positive psychology. Considering that most previous studies discussed PsyCap primarily within the industrial-organizational context, the findings of this study contribute to our understanding of the role of leisure activities in promoting an individual’s positive psychological development and subjective well-being.

## Practical Implication

Our results suggest that when people are experiencing high levels of stress, community involvement has no effect regardless of generation. In this regard, continued efforts are needed to prepare and build a policy or program that can reduce people’s stress. At the same time, our results show that community involvement is effective when people have normal or low levels of stress, highlighting the role of community involvement in increasing PsyCap and life satisfaction. This information indicates that developing effective community engagement strategies that stimulate people’s interest and enthusiasm, while also generating a sense of community involvement, is crucial. More practically, it would be valuable for practitioners or managers in the sport community to use a variety of communication channels (on- and off-line) to increase fans’ social interaction. Not only can the boundaries of the community expand through fan access channels, but the life satisfaction of the fans previously involved in the community can also be boosted for a positive social capital. Taking a more careful look at the generation differences, it may be important to activate various alternative channels for Gen Z as they are known to be omnipresent online (Wood, 2013; Turner, 2015).

Gen Z showed stronger effects of sport community involvement on PsyCap when distress level was lower, compared to the non-Gen Z group. This is consistent with the viewpoint that Gen Z are the most vulnerable to stress (Turner, 2015) while being the least resilient to stress among all generations (Cartwright-Stroupe and Shinners, 2021). Practitioners must acknowledge the differences across generations to prepare for future contingencies. Whereas Gen Z were more distressed during the outbreak, those able to cope with the situation were able to leverage community involvement to boost their PsyCap. Together, these findings elucidate the importance of developing targeted community engagement strategies that take Gen Z’s characteristics into account. For instance, since Gen Z is social and adept at handling technology (Turner, 2015), developing community activities that reflect these characteristics is necessary to generate positive psychological outcomes as well as to reduce their stress.

Practitioners should also focus their attention on establishing and promoting community activity programs that can enhance fans’ PsyCap. Our results showed that fans’ life satisfaction did not increase by the direct effect of sport community involvement but PsyCap mediated to yield positive outcomes. Therefore, the promotion of a program that allows fans to learn and grow as well as be entertained will create a beneficial community (McLaughlin-Volpe et al., 2005). Furthermore, if practitioners or managers of the sport community can develop a program that leads Gen Z to become more involved and engaged, then the sport community will be able to play a positive role as a social connection for Gen Z in difficult times, such as during the pandemic.

Applying the practical implications of our findings to the real world, it will be necessary to develop communication messages and promotional content that members of Gen Z, who are vulnerable to stress and prefer to form like-minded communities, can easily access and handle. Specifically, a strategy that facilitates fan community activities in new communicational environments such as the metaverse and other virtual worlds can be effective to the younger generation (Lee et al., 2021). Based on the self-expansion theory, virtual identities in stress-free environments could engage the younger generation seeking psychological revitalization and varieties. Technology-mediated objects and content such as non-fungible tokens (NFTs) and social memes disseminated through social media could be effective communication tools within sports fan communities. These methods can be applied not only to stress-sensitive members of Gen Z but also to their contemporaries with higher stress tolerances. Developing new content should include elements that Gen Z members can casually consume without being a stressor. For example, recent efforts by the NBA to use the metaverse as an additional viewing channel exemplifies how technology is being used to engage Gen Z fans. Aligning technology-based communication tactics with the current findings, Gen Z members’ involvement in the online and offline communities warrants academic and managerial attention as such experiences can lead to more profound life satisfaction. Involved sport fans will not only merely participate in sports community activities but will acquire knowledge and share their experiences to co-create the community.

As shown in Table 2, the three-way disordinal interaction indicates that Gen Z members’ level of distress exerts a relatively extreme effect on their PsyCap and life satisfaction, compared to the non-Gen Z group. In this regard, practitioners should devise strategies to address each of the conditions under which Gen Z members are expected to be under—either high or low levels of stress. Given that each member of Gen Z faces different levels of stress, customized direct contact messages, which are frequently used in professional sports promotions, may be an effective strategy. Now utilizing data analytic approaches dynamic contact algorithms acquire personal data through previous interactions and customize information based on individual users rather than generic audience characteristics. Such analytic solution provides practitioners with tailored approaches for each sports fan, engaging individuals with a customized selection of information and activities. Thus, practitioners can use such technology to deliver messages which will capture the attention of each individual Gen Z member and provide information while moderating or reducing their stress levels (i.e., pull strategy) rather than increasing stress as a spam message does (i.e., push strategy). Future studies could investigate algorithms to monitor and capture users’ moods and distress.

## Limitations and Suggestions

Several factors may limit the generalization of this study’s findings. Our study investigated a specific context: the sport community. The generalizability of our findings may be limited to different communities, such as those based on celebrities, politics, race, or gender. In future studies, therefore, if we compare and analyze various contexts, we can reveal a wider scope of how community involvement affects an individual. Next, due to the participants in our study skewing toward males more than females, generalizing our findings may be limited. This may be problematic in that there can be gender differences in associations between community activity and its outcomes (Ferris et al., 2013). We expect that a more valid result can be elicited by similarly examining the gender ratio; a more insightful result can be presented by comparing the differences between males and females. Lastly, generations other than Gen Z were not indicated in this study. Although the target generation of this study was Gen Z, various practical implications could be determined if other generations were investigated individually, as each generation has its own distinct characteristics (Seaman et al., 2018). Therefore, we propose to categorize, compare, and analyze other generations in a follow-up study.

## Data Availability Statement

The raw data supporting the conclusions of this article will be made available by the authors, without undue reservation.

## Ethics Statement

The studies involving human participants were reviewed and approved by Texas A&M Institutional Review Board. The patients/participants provided their written informed consent to participate in this study.

## Author Contributions

All authors listed have made a substantial, direct, and intellectual contribution to the work, and approved it for publication.

## Funding

A part of this research was supported by Grant-in-Aid for Early-Career Scientists of Japan Society for the Promotion of Science [JSPS KAKENHI Grant Number JP20292674].

## Conflict of Interest

The authors declare that the research was conducted in the absence of any commercial or financial relationships that could be construed as a potential conflict of interest.

## Publisher’s Note

All claims expressed in this article are solely those of the authors and do not necessarily represent those of their affiliated organizations, or those of the publisher, the editors and the reviewers. Any product that may be evaluated in this article, or claim that may be made by its manufacturer, is not guaranteed or endorsed by the publisher.

## Appendix

Wording of scale items.

**Table:**

Construct/Scale type	Items
Involvement/	1. Unimportant/Important
Semantic differential	2. Boring/Interesting
	3. Irrelevant/Relevant
	4. Unexciting/Exciting
	5. Means nothing to me/Means a lot to me
	6. Unappealing/Appealing
	7. Mundane/Fascinating
	8. Worthless/Valuable
	9. Uninvolving/Involving
	10. Not needed/Needed
Psychological Capital/	1. I always look on the bright side of things regarding my future.
Likert-type	2. I’m optimistic about what will happen to me in the future.
	3. Overall, I expect more good things to happen to me than bad.
	4. I can think of many ways to get out of a jam.
	5. Right now I see myself as being pretty successful.
	6. I can think of many ways to reach my goals.
	7. I feel confident about my ability.
	8. I am able to achieve my goals.
	9. I am capable of handling things in my life.
	10. I tend to bounce back quickly after hard times.
	11. I usually come through difficult times with little trouble.
	12. It does not take me long to recover from a stressful event.
Distress/	1. Feeling no interest in things
Likert-type	2. Feeling lonely
	3. Feeling blue
	4. Feeling of worthlessness
	5. Feeling hopeless about the future
	6. Thoughts of ending my life
	7. Nervousness or shakiness inside
	8. Feeling tense or keyed up
	9. Suddenly scared for reason
	10. Spells of terror or panic
	11. Feeling so restless you could not sit still
Life Satisfaction/	1. In most ways, my life is close to my ideal.
Likert-type	2. The conditions of my life are excellent.
	3. I am satisfied with my life.
	4. So far, I have gotten the important things I want in life.
	5. If I could live my life over, I would change almost nothing.

Items used 7-point format. Likert-type ranged from strongly disagree to strongly agree.
